# Draft Genome Sequence of a Non-O1/O139 Vibrio cholerae Strain Isolated from a Patient Presenting with Dysuria

**DOI:** 10.1128/MRA.00883-18

**Published:** 2018-07-26

**Authors:** Lex E. X. Leong, David L. Gordon, Geraint B. Rogers

**Affiliations:** a Infection and Immunity, South Australian Health and Medical Research Institute, Adelaide, South Australia, Australia; b College of Medicine and Public Health, Flinders University, Bedford Park, South Australia, Australia; c Infectious Diseases Department, Flinders Medical Centre, Bedford Park, South Australia, Australia; d SA Pathology, Bedford Park, South Australia, Australia; Queens College

## Abstract

Vibrio cholerae is the causative agent of cholera and, more rarely, nondiarrheal opportunistic infections. We report here a draft genome sequence of a non-O1/O139 V. cholerae strain isolated from the urine of a patient presenting with dysuria at a South Australian health care facility. The genome analysis revealed several virulence factors important for its pathogenesis.

## Abstract (precis)

Vibrio cholerae is the causative agent of cholera and, more rarely, nondiarrheal opportunistic infections. We report here a draft genome sequence of a non-O1/O139 V. cholerae strain isolated from the urine of a patient presenting with dysuria at a South Australian health care facility.

## ANNOUNCEMENT

Vibrio cholerae, a Gram-negative flagellated bacterium naturally found in saltwater, is the causative agent of cholera. Its ability to cause life-threatening diarrhea is a major public health concern. Although more than 200 V. cholerae serogroups have been described, those associated with epidemic and pandemic cholera are limited to the O1 and O139 serogroups (1). Nevertheless, nonepidemic V. cholerae serogroups, collectively referred to as non-O1/O139 serogroups, can be associated with other less severe infections ranging from cholera-like diarrhea to urinary tract infections (2). In high-income countries, such as Australia, cholera cases caused by epidemic V. cholerae strains are rare; the pathogens are mostly imported from areas of endemicity and typically involve non-O1/O139 V. cholerae strains (3).

We report here the genome sequence of a non-O1/O139 V. cholerae strain isolated from the urine of a 35-year-old female patient of Bangladeshi origin who presented with symptoms of urinary tract infection at a South Australian health care facility. The patient had been residing in South Australia for more than 6 months prior to experiencing a 3-day period of dysuria. Urine microscopy showed pyuria, and a culture identified a Vibrio species that was sensitive to ampicillin, cephalexin, nitrofurantoin, and trimethoprim.

The whole-genome amplicon libraries were prepared using a Nextera XT DNA sample preparation kit (Illumina, Inc., CA, USA) and sequenced on a NextSeq platform with a NextSeq 500/550 mid-output kit (Illumina; V2 chemistry, 2 × 150-bp cycles). A total number of 972,122 paired-end quality-filtered reads were generated (34× coverage against V. cholerae O1 biovar El Tor strain N16961, GenBank assembly accession number GCF_000006745) and assembled using MEGAHIT version 1.1.1 (4). The total size of the draft genome assembly was 3,918,007 bp, with an *N*_50_ of 170 kb, arranged into 95 contigs. The G+C content was determined to be 48%.

Multilocus sequence typing (MLST) analysis with the PUBMLST Vibrio database identified this isolate with a unique sequence type, ST618. Concatenated sequence alignment analysis of the eight MLST alleles showed the closest relative to be derived from environmental sources in China (ST374 and ST402) and Africa (ST343).

The virulence factors linked to these choleragenic V. cholerae strains are a lysogenic filamentous bacteriophage, CTXΦ, encoding for the cholera toxin and the toxin coregulated pilus pathogenicity island (5). Non-O1/O139 V. cholerae strains generally lack these pathogenicity islands but harbor other types of virulence factors, such as hemolysin and non-O1 heat-stable enterotoxin (6, 7). The filamentous bacteriophage CTXΦ and Vibrio pathogenicity islands were not detected in the draft genome of this isolate, but genes encoding the MARTX cholera toxin (*rtxACBD*), hemolysin protein (*hcp2* and *hlyA*), adherence factor IlpA, and flagellar biosynthesis flip were identified. Other virulence genes, such as the type VI secretion system (*vasABCDEFGHIJKL* ), T6SS sheath proteins VipA-VipB, and *S*-ribosylhomocysteine lyase (*luxS*), were also detected in the draft genome. This non-O1/O139 V. cholerae draft genome is a useful resource for future study into V. cholerae virulence factors and evolution.

### Data availability.

This whole-genome shotgun project has been deposited in DDBJ/ENA/GenBank under the accession number QKWT00000000. The version described here is the first version.
